# Expression of angiogenic markers in jawbones and femur in a rat model treated with zoledronic acid

**DOI:** 10.1186/s13104-021-05900-5

**Published:** 2022-01-10

**Authors:** Jing Wen Li, Jing Yi Wang, Ru Qing Yu, Lei Huo, Li Wu Zheng

**Affiliations:** 1 Discipline of Oral and Maxillofacial Surgery, Faculty of Dentistry, The University of Hong Kong, Hong Kong SAR, China; 2 Department of Orthodontics, The First Affiliated Hospital of Zhengzhou University, Zhengzhou, Henan Province China

**Keywords:** Medication-related osteonecrosis of the jaw, Zoledronic acid, Animal model, Gene expression, Angiogenic markers

## Abstract

**Objectives:**

This study aimed to investigate the gene expression of angiogenic marker in surgically treated jawbones and femur on a rat model administrated with zoledronic acid.

**Results:**

No soft tissue fenestration or bone exposure was found in femur. Delayed soft tissue healing was found in both ZA group (3 in mandible, 4 in maxilla) and control group (1 in mandible, 2 in maxilla), while exposed bone was found only in the ZA group (1 in maxilla, 2 in mandible). RT-PCR analysis demonstrated no significant difference in gene expression of angiogenetic markers between ZA-treated and control groups in femur and mandible. In the maxilla, the expression of VEGFA and VEGFR-2 in medium-term ZA group was significantly down-regulated compared with that in the control. The ZA treatment does not change significantly the expression of the angiogenic factors in femur and mandible, but significantly downregulates the expression in maxilla in this rat model. The angiogenesis inhibition may contribute to the development of MRONJ but does not play a key role.

**Supplementary Information:**

The online version contains supplementary material available at 10.1186/s13104-021-05900-5.

## Introduction

Bisphosphonates (BPs) have been extensively used for management of bone diseases with pathologically high resorption. Despite the great clinical benefits, a severe complication known as medication-related osteonecrosis of the jaw (MRONJ) has been reported [1]. The potentially compromised neovessel formation in the development of MRONJ has been investigated but the results remain controversial.

Vascular endothelial growth factor A (VEGFA) plays a critical role in angiogenesis, promotion of vessel permeability and also participates in the processes of bone tissue formation, healing and remodeling [2–5]. VEGFA binds to target cell receptors such as VEGF receptor (VEGFR)-1, -2 and -3. At binding, VEGFA activates these receptors in extracellular space (ECs) and promotes transduction of different signals in EC migration, proliferation and enhancement of angiogenesis [6, 7]. VEGFR-2 is an essential mediator of VEGF-initiated angiogenesis. Levels of VEGFR-2 expression is relatively low in adult blood vessels, however, it is markedly upregulated in blood vessels in some pathological conditions, such as chronic inflammation, wound repair and cancer [8–11]. CD31 is a marker of vascularization inclusive of microvessels [12–14]. S. Ishtiaq et.al investigated the circulating concentrations of VEGF and angiopoietin-1 (ANG-1) in post-menopausal women receiving alendronate therapy, and with osteoblastic production of VEGF and ANG-1 under zoledronate and alendronate treatments in vitro. [15] The results showed that BPs suppressed osteoblastic production of angiogenic factors in vitro and in vivo, indicating the possible relevance of angiogenesis inhibition in MRONJ pathophysiology. However, other studies revealed an upregulation of angiogenic factors after long-term BPs treatment, which might be a result of compensation for BPs’ antiangiogenic potency [16–18]. Interestingly, levels of serum markers of angiogenesis and inflammation were also found to be higher in MRONJ patients after cession of long-term BPs therapy [17]. Higher expression of VEGFA in postoperative exudate of bisphosphonate-related osteonecrosis of the mandible was also reported [19]. There are reports showed decreased expression of VEGF in oral mucosa in patients receiving BPs treatment without MRONJ lesions, and a massive increase of VEGF in MRONJ mucosal lesions [20]. The contradictory findings suggested a possible alteration of angiogenesis in the development of MRONJ. The evidence is still lacking for the angiogenic character of this pathological condition.

This study aimed to investigate the gene expression of angiogenic markers in surgically treated jawbones and femur using a rat model administrated with zoledronic acid administration.

## Main text

### Material and methods

#### Animal care and surgery

Thirty-two 12-week-old female Sprague–Dawley (SD) rat were obtained from the Laboratory Animal Unit of Li Ka Shing Faculty of Medicine, The University of Hong Kong. The protocol of the animal experiment was approved by the Committee on Use Live Animal for Teaching and Research, The University of Hong Kong (CULATR 3775-15). The animals were housed in an indoor environment at a temperature of 20 °C ± 5° in a 12:12-h light–dark circle with free access to water and standard rodent diet (Irradiated, PMI, USA).

Animals were general anesthetized intraperitoneally with a mixture of 67 mg/kg ketamine and 6 mg/kg xylazine (Alfasan International B.V., Woerden, Holland). The right thigh was shaved and sterilized with 10% betadine solution. A 1.5-cm-long incision was made followed with fascia and muscle dissection. Femer was completely exposed after periosteum exfoliation. A unicortical circular defect was created at the lateral aspect of femer with a round bur using a Ø2.0 trephine bur (Trephine Drill 2 mm (3 mm OD) × 10 mm Barrel, ForevreGreen, Hong Kong) by low-speed handpiece without any further invasion to bone marrow. Surgical site was continuously irrigated with sterile saline solution to avoid contamination and overheat. Cortical bone layer was then removed and bone marrow was exposed. The soft tissues were closed in two layers. The muscular layer was sutured using 4-0 vicryl (Ethicon Inc., Cornelia, GA), the skin was sutured using 5-0 Prolene (Ethicon Inc., Cornelia, GA). Following the right femur operation, the right first maxillary and mandibular molars were extracted using a standard protocol. Two gauze rolls and a retractor were used to maintain the mouth open and to make the tongue immovable. After gingiva separation with dental explorer the molars were carefully removed using children’s extracting forceps, in order to avoid any damage to surrounding tissue. The extraction sites were left open and a small cotton wool roll was pressed onto the extraction socket until bleeding stops.

#### Grouping and treatment

Sixteen rats received zoledronic acid treatment (ZA group), and the rest rats serving as the control group received saline solution. Eight rats in each group were sacrificed at 2 weeks (short-term group) and 4 weeks (medium-term group) after surgery, respectively.

From four weeks before surgery until sacrifice, animals in ZA groups were given 66 µg/kg of zoledronic acid (Zometa, Novartis, Switzerland) intraperitoneally three times per week. In control group, the same volume of normal saline solution was administered with the same timing protocol.

#### Sacrifice, sample collection and preparation

Pentobarbital sodium (Dorminal^®^, Alfasan International B.V., Woerden, Holland) was administered at a dose of 150 mg/kg intraperitoneally for animal sacrifice. Euthanasia was confirmed by observing that there were no respiratory movement at least three minutes and heartbeat had ceased. Gross observations of the samples were performed, and photo records were taken.

The jawbones and right femur were removed with soft tissue stripped off. A rongeur was used to collect the bone tissue in the extraction socket and femur defect. The collected tissue was immediately stored in RNAlater^®^ solution (AM7024, Life Technologies Limited, California, United States) and stored at − 20 °C for further assessments.

#### RNA extraction and cDNA synthesis

The total RNA from femur defect site and jawbones tooth extraction site in both control and ZA-treated samples were extracted using RNeasy Lipid Tissue Mini Kit (QIAGEN, Hilden, Germany). The total RNA concentrations were quantified using Nanodrop ND-2000c spectrophotometer (Thermo Fisher Scientific, Massachusetts, US) at 260 nm, and non-contamination with proteins was verified according to the 260/280 ratio. The SuperScript III Reverse transcriptase (Invitrogen Corporation, US) was used to synthesize complementary fxdeoxyribonucleic acid (cDNA) in a PCR thermal cycler (GeneAmp PCR System 9700, Thermo Fisher Scientific, Massachusetts, US).

#### Real-time polymerase chain reaction (RT-PCR) analysis

Quantitative real-time PCR (RT-PCR) was performed using FAST SYBR™ Green Master Mixes (Applied Biosystems™, Hercules, CA, USA) in ABI Prism 7000 sequence detection system (Applied Biosystems™, Hercules, CA, USA) following manufacturer’s instruction.

All samples were run in MicroAmp™ Fast Optical 96-well reaction plates. The reactions conditions were performed at 95 °C for 20 s, following with 40 cycles of 95 °C for 3 s and 60 °C for 30 s. Four pairs of primers, including CD31, VEGFA, VEGFR-2 and housekeeping gene GAPDH, were designed using Primer Express software (Applied Biosystems™, Hercules, CA, USA) (Table 1). Relative quantitation of gene expression was assessed between quantity of target gene and GAPDH using relative standard curve method.

**Table 1:** Primers designed for amplifying target and housekeeping genes in RT-PCR

Gene	Forward primer (5′-3′)	Reverse primer (5′-3′)
CD31	TGGAAACCAACAGCCATTACG	GGGAGCCTTCCGTTCTCTTG
VEGFA	CAGGAGCCTGGCCATCAA	CCTCTTCTTCCACCACTGTGTCT
VEGFR-2	TGGTGGCTCAGGACGTTGA	TTCCCCTTTCTCCTCCGTTT
GAPDH	GGTGGACCTCATGGCCTACA	CAGCAACTGAGGGCCTCTCT

The expression values of CD31, VEGFA and VEGFR-2 were normalized to the expression of GAPDH. A standard curve was carried out using the target DNA which subjected to a serial dilution. The signals in the sample were within the cycles covered by the standard curve. A melting curve was carried out to indicate the quality of the product, in which a high-quality product gave a narrow peak when denatured.

PCR data analysis was performed using StepOne™ software v2.0.2 (Thermo Fisher Scientific, Waltham, US). Relative quantitation of gene expression was assessed between quantity of target gene and GAPDH using relative standard curve method.

#### Statistical analysis

IBM SPSS 24.0 (IBM Crop, Armonk: NY, USA) was used for data analyses. Relative expression levels of genes were expressed as mean ± standard deviation and statistically analyzed using independent-samples *t*-test. P < 0.05 was considered statistically significant.

### Results

#### Clinical observation

Gross observation of defect site showed normal healing of surgical wounds in majority (28/32) of the animals in ZA and Control groups (Additional file 1: Figs. S1, S2). No soft tissue fenestration or bone exposure was found in femur. Delayed soft tissue healing was found in both ZA group (3 in mandible, 4 in maxilla) and control group (1 in mandible, 2 in maxilla), while exposed bone was found only in the ZA group (1 in maxilla, 2 in mandible) (Table 2).

**Table 2:** Anatomy observation in the mandibular, maxillary extraction site and femur defect

	N	Femur		Mandible		Maxilla	
		Soft tissue fenestration	Exposed bone	Soft tissue fenestration	Exposed bone	Soft tissue fenestration	Exposed bone
Treatment	16	0	0	3 (19%)	2 (13%)	4 (25%)	1 (6%)
ZA-s	8	0	0	1 (13%)	0	3 (38%)	0
ZA-m	8	0	0	2 (25%)	2 (25%)	1 (13%)	1 (13%)
Control	16	0	0	1 (6%)	0	2 (13%)	0
C-s	8	0	0	1 (13%)	0	1 (13%)	0
C-m	8	0	0	0	0	1 (13%)	0

#### Real-time PCR

All the animals were sacrificed for real-time PCR analysis to determine the relative mRNA expression levels of angiogenesis markers CD31, VEGFR-2 and VEGFA. Serial two-time dilution of the cDNA was applied for RT-PCR. Standard curve for each target gene was obtained (Additional file 1: Figs. S3, S4).

The fold-difference of quantity in ZA group relative to control group was shown in Table 3. In femur defect, the expression of these angiogenetic markers showed no significant difference between control groups and ZA-treated groups. In the mandible, 1.12-fold of VEGFA expression was found in short-term ZA-treated group compared with corresponding control (p = 0.016). Among medium-term groups, expression of CD31, VEGFA and VEGFR-2 were higher in ZA-treated group but without statistically significance. In the maxilla, short-term ZA group expressed 1.43-fold of VEGFR-2 expression relative to the corresponding control (p = 0.04). While medium-term ZA group showed 2.4-fold of CD31 expression compared with control (p = 0.048). The expressions of VEGFA and VEGFR-2 were down-regulated in medium-term ZA group, in which 0.41-fold of VEGFA (p = 0.029) and 0.73-fold of VEGFR-2 (p = 0.039) lower than their corresponding control (Additional file 1: Fig. S5).

**Table 3:** The fold-difference between ZA-treated group normalized target and control group normalized target

Targets	Femur		Mandible		Maxilla	
	Short-term	Medium-term	Short-term	Medium-term	Short-term	Medium-term
CD31						
*Fold*^a^	1.02 ± 0.21	1.15 ± 0.79	0.69 ± 0.40	2.39 ± 1.13	1.32 ± 0.97	2.40 ± 0.67
*p*	0.880	0.770	0.270	0.230	0.590	**0.048**
VEGFA						
* Fold*	0.99 ± 0.15	1.28 ± 0.61	1.12 ± 0.43	1.45 ± 0.18	1.33 ± 0.30	0.41 ± 0.10
*p*	0.120	0.820	**0.016**	1.000	0.060	**0.029**
VEGFR-2						
*Fold*	1.38 ± 0.24	0.87 ± 0.42	1.37 ± 0.54	1.19 ± 0.10	1.43 ± 0.54	0.73 ± 0.14
*p*	0.480	0.400	0.069	0.082	**0.040**	**0.039**

Differences are considered significant at p < 0.05 and highlighted in bold

^a^Fold-difference of ZA-treated group relative to control group, all values are normalized to GAPDH expression

### Discussion

MRONJ is realized as a pathological osteonecrosis condition of craniofacial skeleton with significant symptom of bone exposure after administration of antiresorptive agents or anti-angiogenesis drugs despite the existence of radiation treatment history. In our recent study, which used the same animal model, found that zoledronic acid treatment had a site-specific effect on surgically treated jawbones versus long bones, where necrosis occurred only in the jaw. This could be explained by the theory that Bps were considered as more preferentially deposited in skeleton structures with high turnover so that jawbones were more likely to be affected by these drugs. At the same time, osteonecrosis occurred more frequently at the site of the mandibles than maxillae since blood supply could play an important role to the metabolic activity [21]. The present study demonstrated similar result. No soft tissue fenestration or bone exposure was found in femur. Exposed bone was found only in the ZA treated animals, 1 out of 16 rats (6%) in the maxillary extraction site, and 2 out of 16 (13%) in the mandibular extraction site, indicating positive relevance of ZA treatment and osteonecrosis in jawbones.

The development of MRONJ was realized as multifactorial including osteoclast suppression, immune alteration, macrophage change, anti-angiogenesis, cell accumulation et al. In this study, we focused on the research of angiogenesis expression of animal model with BPs treatment. According to previous studies, it had been well known that BPs decrease endothelial proliferation which may influence angiogenesis [22, 23]. A micro-CT study performed by our group on the same rat model revealed that the ZA treatment had no significant effect on the microvasculature structure in the surgical healing sites of the jawbones and femur. A tendency of decreased vessel density and vessel number in ZA-treated group was found, but no statistical difference [24]. Inconsistent with the micro-CT findings, the present study found the expression of CD31, VEGFA and VEGFR-2 in the ZA treated group was slightly higher than that in the control group in femur, but no statistical significance. Similar results were found in the mandible, with the exception that VEGFA expression in the short-term ZA group was significantly higher (1.12-fold) when compared to the control group. The result indicated that in this study, the administration of ZA did not affect blood vessel formation. This was also reported by other studies which claimed normal angiogenesis in ONJ lesions [25, 26]. The upregulated tendency of angiogenetic markers in the femur and mandible, which is inconsistent with previous in vitro, animal and clinical studies, might be a compensatory result of the antiangiogenic potency of BPs [16–18].

Interestingly, the present study demonstrated a different expression pattern of the angiogenetic markers in maxilla. The gene expression of VEGFA and VEGFR-2 in ZA-treated group is significantly down-regulated compared with control. The unexpected finding in maxilla might be attributed to the difference in anatomical and angiogenic structures between mandible and femur, however, the down-regulated expression of the angiogenic factors in maxilla in the ZA treated group does not result in a compromised angiogenesis [27, 28].

Overall, the expression of the angiogenic factors in femur and mandible is not significantly affected by BPs treatment, whereas it is significantly decreased in response to surgical intervention in maxilla. Given the higher occurrence of MRONJ in mandible compared with that in maxillary bone, the down-regulated expression of angiogenic factor under ZA treatment may not be directly associated with the site-specific feature of MRONJ.

### Conclusions

The ZA treatment does not change significantly the expression of the angiogenic factors in femur and mandible, but significantly downregulates the expression in maxilla in this rat model. The angiogenesis inhibition may contribute to the development of MRONJ but does not play a key role.

### Limitation

It is noteworthy that this study was primarily focused on the assessment of bone exposure and osteonecrosis, however, with the limitation of the known factors, more investigations are required for more understanding of the underlying mechanisms responsible for the markers of angiogenesis in soft tissue such as gums of the animals treated with zoledronic acid.

## Supplementary Information

**Additional file 1: Figure S1.** Gross observation of healing of the extraction site on right maxillary first molar in ZAtreated groups and control groups. ZAs: Delayed soft tissue healing could be observed (yellow arrow); ZAm: Suspicious of bone exposure on the site of the extraction socket (yellow arrow); ZAl, Cs, Cm Cl: Optimal healing of soft tissue on the extraction site. Blue arrows indicate normal healing after tooth extraction. M2: Maxillary second molar. **Figure S2.** Gross observation of healing of the extraction site on right mandibular first molar in ZA-treated groups and control groups. ZAs: Delayed soft tissue healing on the extraction site (yellow arrow); ZAm: Suspicious infection and bone exposure on the extraction area (yellow arrow); ZAl: Suspicious bone exposure on the extraction site (yellow arrow); Cs: Slightly delayed healing of soft tissue (yellow arrow); Cm, Cl: Optimal healing of soft tissue on the extraction site (blue arrow). M2: Mandibular second molar. **Figure S3.** Amplification plot for housekeeping gene GAPDH (a) and target gene CD31 (c); Standard curve for GAPDH (b), R^2^ = 0.998, and target gene CD31(d). R^2^ = 0.997. R^2^: correlation coefficient. **Figure S4.** Amplification plot for target gene VEGFA (a) and VEGFR-2 (c); Standard curve for VEGFA (b), R^2^ = 0.994, and VEGFR-2 (d). R^2^ = 0.997. R^2^: correlation coefficient. **Figure S5.** Relative quantity of CD31, VEGFR-2 and VEGFA in bone biopsies (femur (a, d, g); mandible (b, e, h); and maxilla (c, f, i)) of 2 weeks and 4 weeks post-operation. Values are normalized to GAPDH expression. Difference is considered significant at *p < 0.05

## Data Availability

The datasets generated and analysed during the current study are available from the corresponding author on reasonable request.

## Abbreviations

BPs: Bisphosphonates
CD31/PECAM-1: Cluster of differentiation 31/platelet endothelial cell adhesion molecule-1
cDNA: Complementary deoxyribonucleic acid
ECs: Extracellular space
GAPDH: Glyceraldehyde 3-phosphate dehydrogenase
MRONJ: Medication-related osteonecrosis of the jaws
RT –PCR: Real-time polymerase chain reaction
SD-rat: Sprague–Dawley rat
VEGF: Vascular endothelial growth factor
VEGFA: Vascular endothelial growth factor A
VEGFR-2: Vascular endothelial growth factor receptor-2
ZA: Zoledronic acid
