# Supplementary material for: Expression of angiogenic markers in jawbones and femur in a rat model treated with zoledronic acid
Source: BMC Res Notes. 2022 Jan 10;15:12. doi: 10.1186/s13104-021-05900-5 (PMC8751108; doi:10.1186/s13104-021-05900-5)
Supplement: Supplementary file 1 — Additional file 1: Figure S1. Gross observation of healing of the extraction site on right maxillary first molar in ZAtreated groups and control groups. ZAs: Delayed soft tissue healing could be observed (yellow arrow); ZAm: Suspicious of bone exposure on the site of the extraction socket (yellow arrow); ZAl, Cs, Cm Cl: Optimal healing of soft tissue on the extraction site. Blue arrows indicate normal healing after tooth extraction. M2: Maxillary second molar. Figure S2. Gross observation of healing of the extraction site on right mandibular first molar in ZA-treated groups and control groups. ZAs: Delayed soft tissue healing on the extraction site (yellow arrow); ZAm: Suspicious infection and bone exposure on the extraction area (yellow arrow); ZAl: Suspicious bone exposure on the extraction site (yellow arrow); Cs: Slightly delayed healing of soft tissue (yellow arrow); Cm, Cl: Optimal healing of soft tissue on the extraction site (blue arrow). M2: Mandibular second molar. Figure S3. Amplification plot for housekeeping gene GAPDH (a) and target gene CD31 (c); Standard curve for GAPDH (b), R2 = 0.998, and target gene CD31(d). R2 = 0.997. R2: correlation coefficient. Figure S4. Amplification plot for target gene VEGFA (a) and VEGFR-2 (c); Standard curve for VEGFA (b), R2 = 0.994, and VEGFR-2 (d). R2 = 0.997. R2: correlation coefficient. Figure S5. Relative quantity of CD31, VEGFR-2 and VEGFA in bone biopsies (femur (a, d, g); mandible (b, e, h); and maxilla (c, f, i)) of 2 weeks and 4 weeks post-operation. Values are normalized to GAPDH expression. Difference is considered significant at *p < 0.05 [file 13104_2021_5900_MOESM1_ESM.pdf]

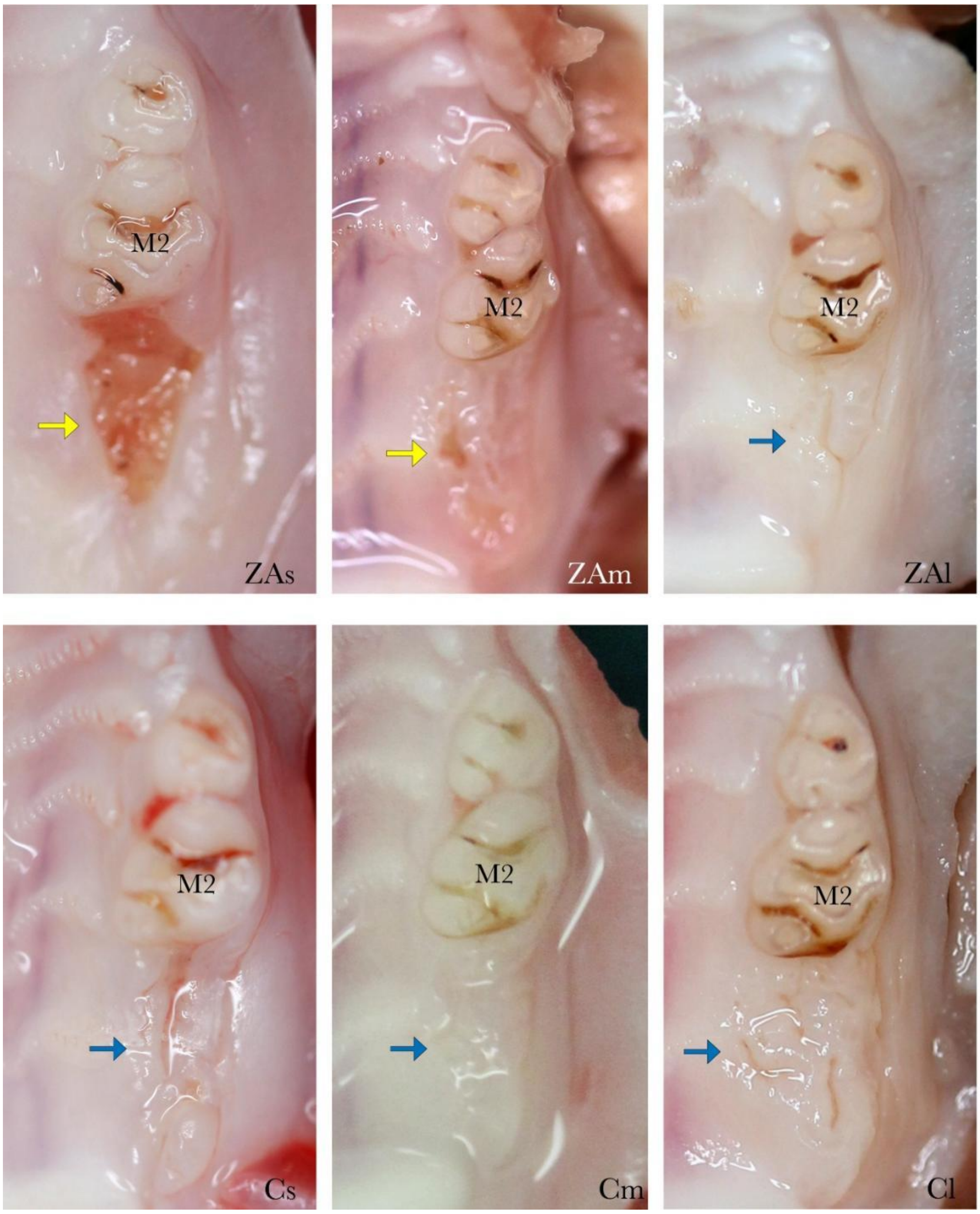

Figure S1 Gross observation of healing of the extraction site on right maxillary first molar in ZA-treated groups and control groups. ZAs: Delayed soft tissue healing could be observed (yellow arrow); ZAm: Suspicious of bone exposure on the site of the extraction socket (yellow arrow); ZAl, Cs, Cm Cl: Optimal healing of soft tissue on the extraction site. Blue arrows indicate normal healing after tooth extraction. M2: Maxillary second molar.

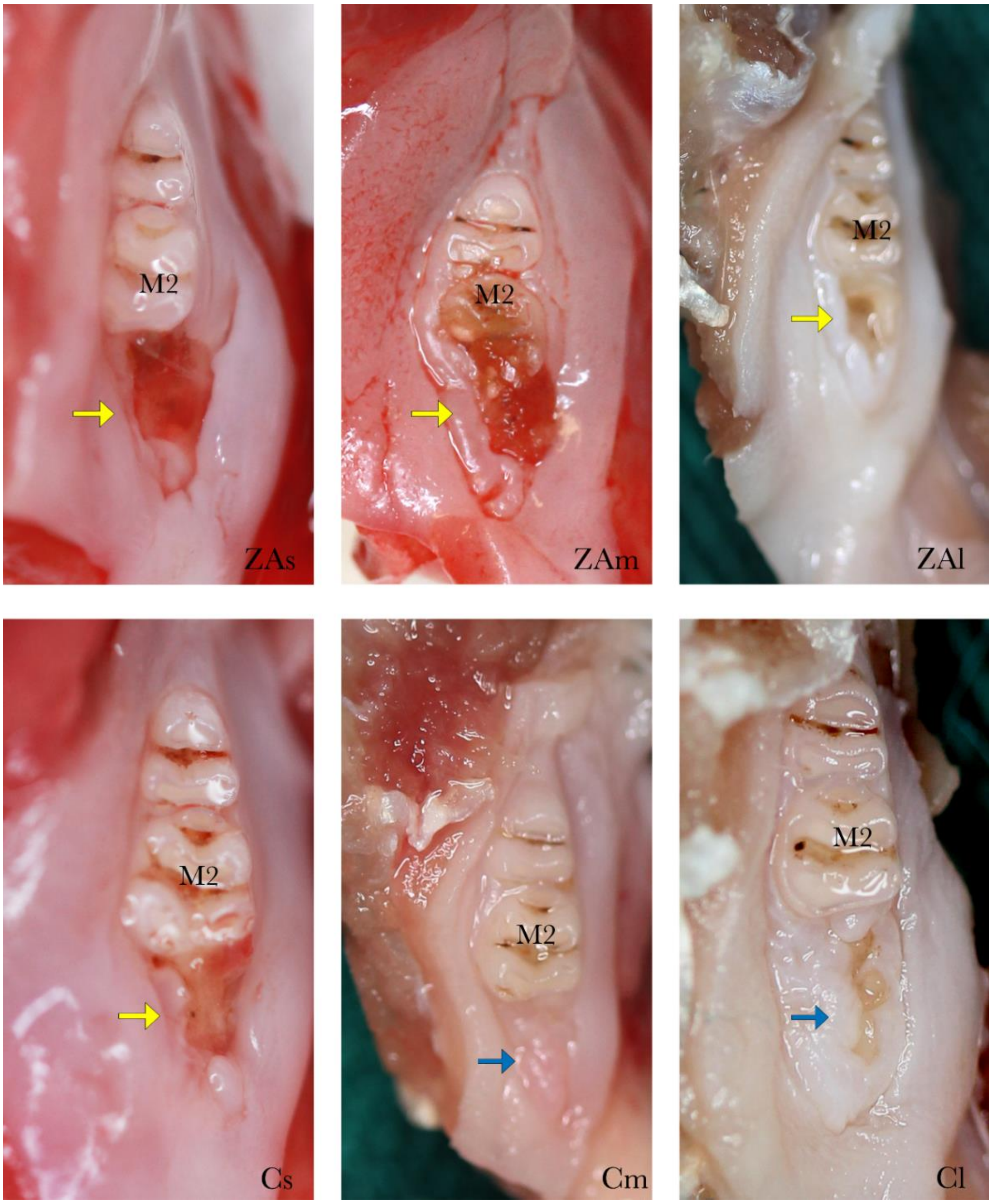

Figure S2 Gross observation of healing of the extraction site on right mandibular first molar in ZA-treated groups and control groups. ZAs: Delayed soft tissue healing on the extraction site (yellow arrow); ZAm: Suspicious infection and bone exposure on the extraction area (yellow arrow); ZAl: Suspicious bone exposure on the extraction site (yellow arrow); Cs: Slightly delayed healing of soft tissue (yellow arrow); Cm, Cl: Optimal healing of soft tissue on the extraction site (blue arrow). M2: Mandibular second molar.

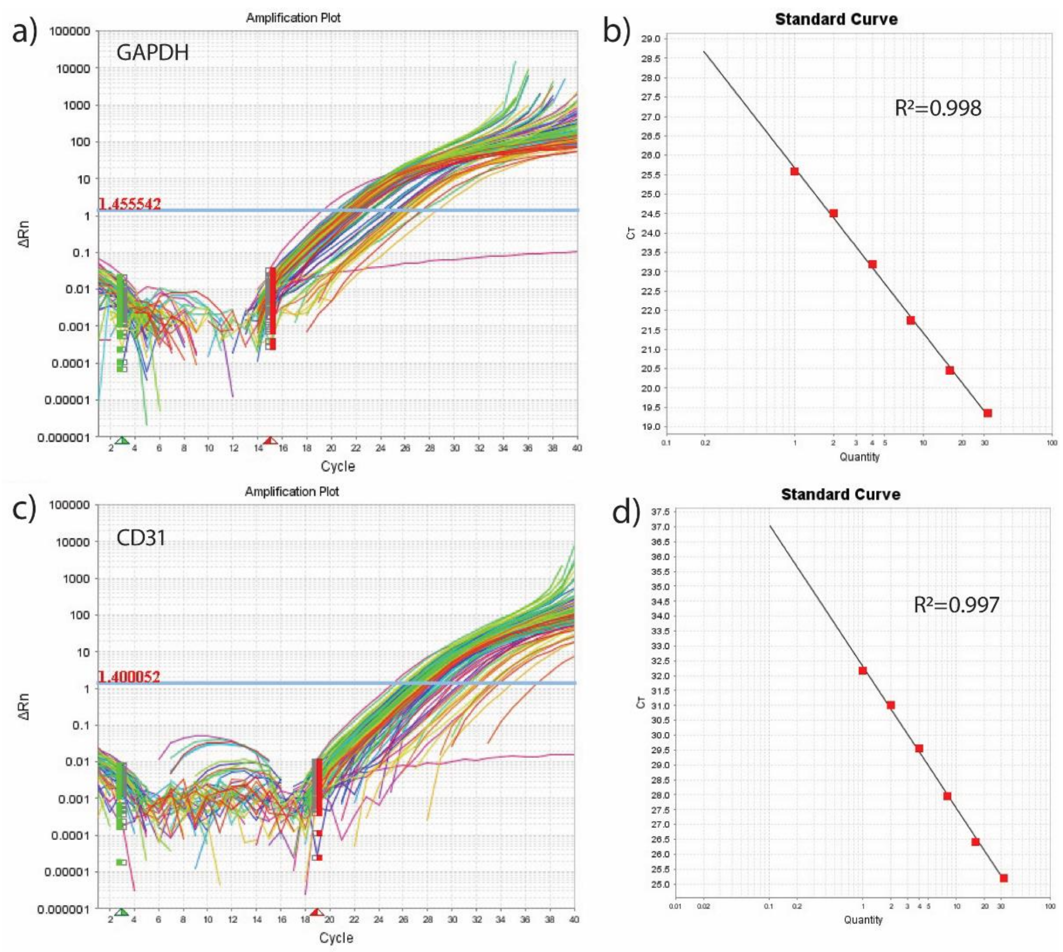

Figure S3 Amplification plot for housekeeping gene GAPDH (a) and target gene CD31 (c); Standard curve for GAPDH (b), R^2=0.998, and target gene CD31(d). R^2=0.997. R^2: correlation coefficient.

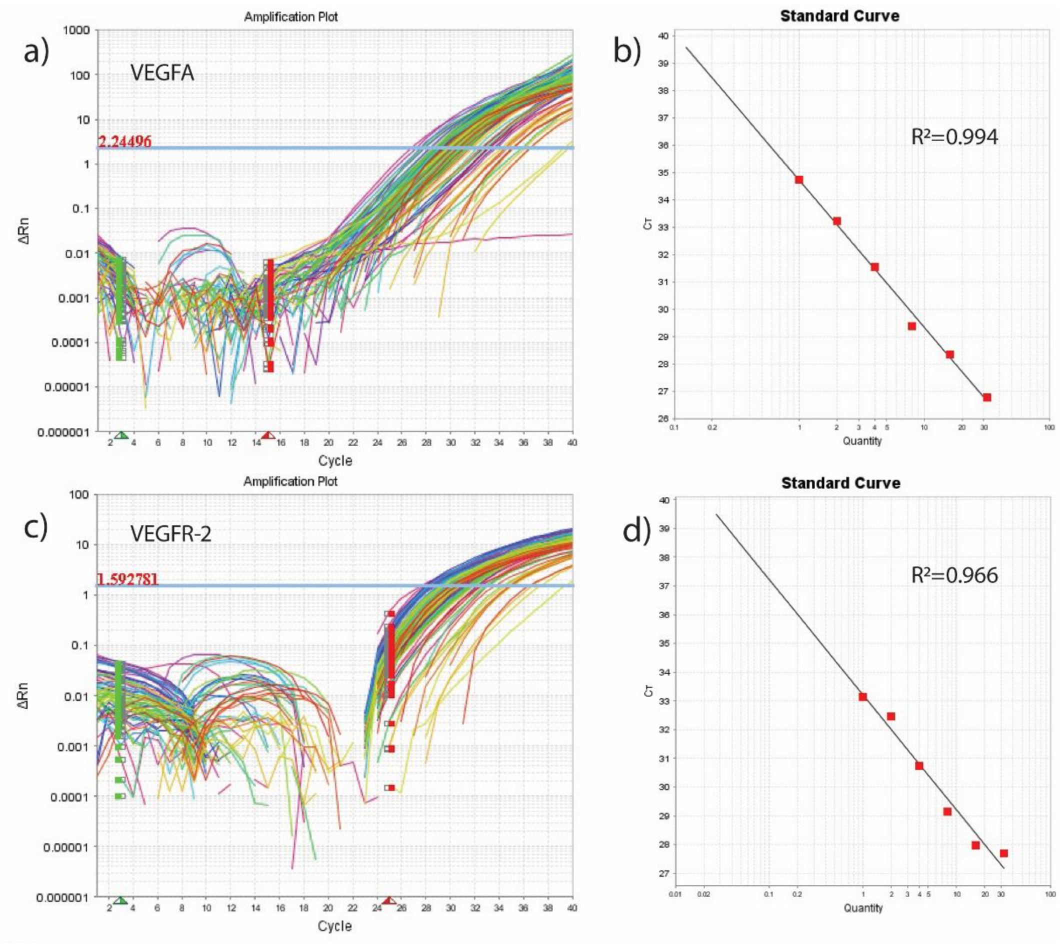

Figure S4 Amplification plot for target gene VEGFA (a) and VEGFR-2 (c); Standard curve for VEGFA (b), R^2 =0.994, and VEGFR-2 (d). R^2 =0.997. R^2: correlation coefficient.

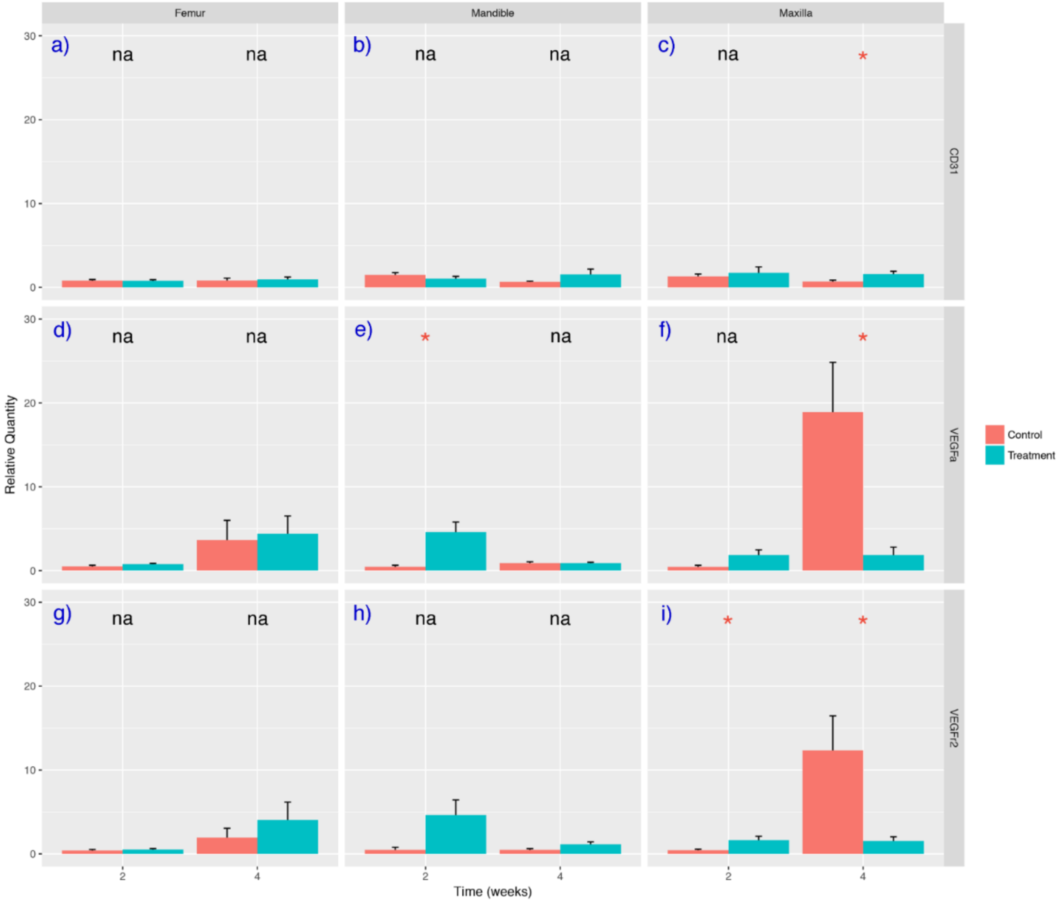

Figure S5 Relative quantity of CD31, VEGFR-2 and VEGFA in bone biopsies (femur (a, d, g); mandible (b, e, h); and maxilla (c, f, i)) of 2 weeks and 4 weeks post-operation. Values are normalized to GAPDH expression. Difference is considered significant at *p<.05
